# Photodynamic antimicrobial chemotherapy with the novel amino acid-porphyrin conjugate 4I: *In vitro* and *in vivo* studies

**DOI:** 10.1371/journal.pone.0176529

**Published:** 2017-05-11

**Authors:** Yao Yuan, Zi-Quan Liu, Heng Jin, Shi Sun, Tian-Jun Liu, Xue Wang, Hao-Jun Fan, Shi-Ke Hou, Hui Ding

**Affiliations:** 1 Institute of Disaster Medicine and Public Health, Affiliated Hospital of Logistic University of the Chinese People’s Armed Police Force (PAP), Tianjin, China; 2 Department of Emergency Medicine, Tianjin Medical University General Hospital, Tianjin, China; 3 Tianjin Key Laboratory of Biomedical Material, Institute of Biomedical Engineering, Peking Union Medical College – Chinese Academy of Medical Sciences, Tianjin, China; Massachusetts General Hospital, UNITED STATES

## Abstract

Photodynamic antimicrobial chemotherapy (PACT), as a novel and effective therapeutic modality to eradicate drug resistant bacteria without provoking multidrug resistance, has attracted increasing attention. This study examined the antimicrobial efficacy of the novel cationic amino acid-porphyrin conjugate 4I with four lysine groups against two different clinical isolated strains (drug sensitive and multidrug resistant) of the *Acinetobacter baumannii* species and its toxicity on murine dermal fibroblasts *in vitro*, as well as the therapeutic effect of PACT on acute, potentially lethal multidrug resistant strain excisional wound infections in vivo. The PACT protocol exposed 4I to illumination, exhibiting high antimicrobial efficacy on two different strains due to a high yield of reactive oxygen species (ROS) and non-selectivity to microorganisms. The photoinactivation effects of 4I against two different strains were dose-dependent. At 3.9 μM and 7.8 μM, PACT induced 6 log units of inactivation of sensitive and multidrug resistant strains. In contrast, 4I alone and illumination alone treatments had no visibly antimicrobial effect. Moreover, cytotoxicity tests revealed the great safety of the photosensitizer 4I in mice. In the *in vivo* study, we found 4I-mediated PACT was not only able to kill bacteria but also accelerated wound recovery. Compared with non-treated mice, over 2.89 log reduction of multidrug resistant *Acinetobacter baumannii* strain was reached in PACT treat mice at 24 h post-treatment. These results imply that 4I-mediated PACT therapy is an effective and safe alternative to conventional antibiotic therapy and has clinical potential for superficial drug-resistant bacterial infections.

## Introduction

The discovery of penicillin was the beginning of the Golden Age of antibiotics in the 1940s, in which penicillin rapidly became a widespread clinical medication. Other antibiotics, such as streptomycin, chloramphenicol and tetracycline, were also isolated soon thereafter. However, because of antibiotic abuse and the rapid evolution of pathogens, increasing numbers of pathogens quickly developed drug-resistance to antibiotics to which they were originally sensitive. Thus, the time of momentous victories against pathogens has passed. Among the large number of antibiotic-resistant pathogenic bacterial species, *Acinetobacter baumannii*, a ubiquitous, aerobic, non-fermentive, opportunistic, Gram-negative coccobacillus, has recently attracted much attention and has been identified as one of the most troublesome pathogens. Because of its particular physical structure, this bacterium is resistant to many antibiotics and survives on artificial surfaces for extended periods of time more easily [1, 2]. In addition, high disease severity, debilitating conditions for patients, long bedridden hospitalizations, long durations of mechanical ventilation, and prior exposure to several antibiotics have made multidrug resistant *Acinetobacter baumannii* emerge at a remarkable rise in intensive care units (ICUs). Further, treatment has been one of the most intractable challenges. High mortality rates, ranging from 38% to 46% despite appropriate treatment or between 17% and 52%, longer total days of admission and average extra total admission cost, are reported in large studies [3–5]. Therefore, the research and development of new drugs without developing resistance is imperative [6, 7]. As a novel and effective therapeutic modality to eradicate drug resistant bacteria without provoking drug resistance, photodynamic antimicrobial chemotherapy (PACT) has recently attracted increasing attention. Similar to photodynamic therapy (PDT), PACT employs non-toxic photosensitizers (PS). PACT kills the microbes by nonspecific oxidative damage by reactive oxygen species (ROS), such as singlet oxygen and hydroxyl radicals, which are produced by the irradiation of the PS with certain wavelengths of light. Additionally, PACT is safe to host-tissue [8].

Porphyrin, a tetrapyrrole backbone macrocycle compounds, was the first PS employed for clinical therapy, which was introduced by Dougherty and co-workers, and its many acknowledged advantages, such as matching absorption wavelength with biotissue, high singlet oxygen yields, and convenient synthesis, led to the universality of its application throughout the world [9–11]. The marked difference in the cell wall structures, chemical composition and charge between Gram-positive and Gram-negative bacteria usually makes the cationic porphyrins more efficient than neutral and negatively charged analogues in the photo inactivation chemical reaction [12, 13]. However, the intrinsic characteristics of the unnatural cationic groups do not endow the derivatives with good biocompatibility, and the uptake by bacteria is limited. As our team previously reported [9], aiming to attenuate the disadvantages, three native basic amino acids, L-lysine, L-histidine and L-arginine, were covalently conjugated with amino porphyrins as cationic auxiliary groups. Thirteen target compounds were synthesized, and compound 4I, an amino phenyl porphyrin bearing four lysine moieties, displayed the highest photo inactivation efficacy against MRSA, *Escherichia coli* and *Pseudomonas aeruginosa*, and decreased cytotoxicity in A929 cells. Therefore, in this work, we investigated the effects of PDT on bacterial inactivation and the wound healing process, using 4I in a mouse cutaneous excisional wound model topically infected with a pan-resistant *A*. *baumannii* strain. Furthermore, the antibacterial activity of 4I against *A*. *baumannii* and the phototoxicity in 3T3 cells were measured *in vitro*.

## Materials and methods

### Photosensitizer & light source

**Fig 1:**
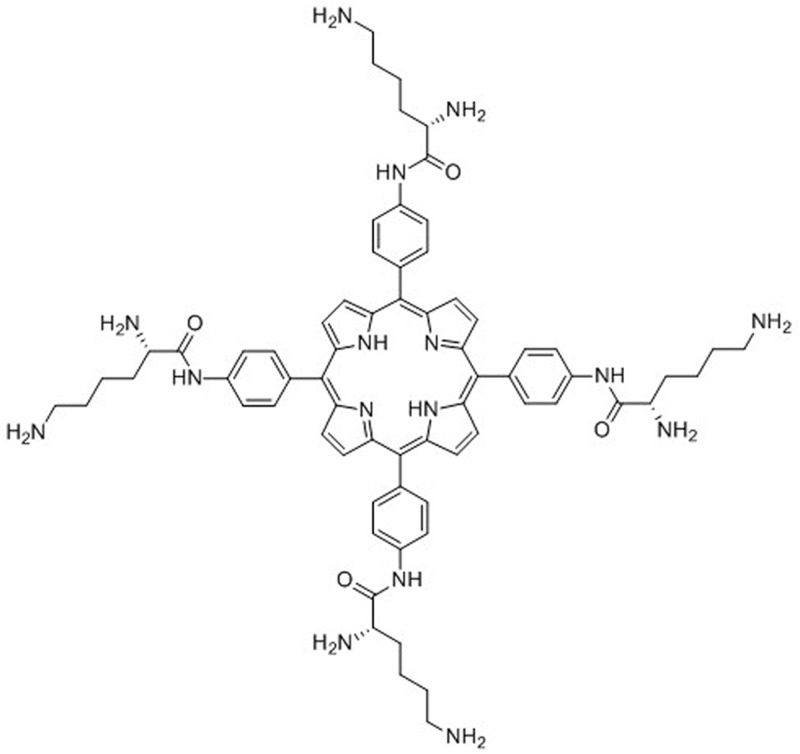
Structural formula of 4I. Molecular formula: 5, 10, 15, 20-trakis(4-((s)-2, 6-diaminohexanamido)-phenyl) porphyrin; molecular weight: 1187.

The PS 4I (Fig 1), a cationic porphyrin conjugate bearing four lysine moieties dissolved in deionized water as a 1000 μM solution, and the light source, a semiconductor laser (7404, Intense, USA) that emits red light at a centre wavelength of 650 nm, were supplied by professor Liu of the Bioengineering Research Institute of Peking Union Medical College. The full synthetic methods and characterization of 4I have previously been described in detail [9].

### Bacterial strains & culture conditions

The bacteria employed in this study were a multidrug-resistant *A*. *baumanni* strain and a relatively sensitive *A*. *baumannii* strain, which were isolated from a cerebral infarction patient in the ICU and a lung cancer patient in the respiratory department, respectively. An antibacterial susceptibility test showed resistance to 14 types of antibiotics, including reference drugs such as ampicillin, cefepime and ciprofloxacin. For experiments, *A*. *baumannii* was grown overnight in Luria Bertani (LB) broth in an orbital shaking incubator (37°C, 120 rpm). The bacterial growth was assessed with a spectrophotometer (Thermo Scientific, Waltham, MA). When optical density (OD) at 600 nm reaches 0.1, which corresponds to a bacterial cell density of 10^8^ CFU/mL. Cells were washed and re-suspended in phosphate-buffered saline (PBS, pH = 7.4) and used at a density of 10^8^ CFU/mL for *in vivo* experiments. The cells were diluted 100-fold in PBS for *in vitro* experiments.

Both a multidrug-resistant and a relatively sensitive *A*. *baumannii* strain were used in this photoinactivation ability evaluation study. 100 μl of bacterial suspension (10^6^ CFU/mL) in PBS and 100 μl of 4I solution (concentrations ranged from 1.95, 3.90, 7.80, 15.60, 31.25, 62.5, 125 and 250 μM, by double dilutions with PBS, and there were three duplicate wells for each concentration) were transferred to wells in a 96-well plate (the definitive effect of 4I at concentrations ranged from 0.975 to 125 μM) and incubated in the dark for 30 min at room temperature with gentle shaking prior to illumination with 650 nm red light, a fluence of 6 J/cm^2^ for 30 min. Attention was paid to ensure that the bacterial suspensions were mixed thoroughly before being placed in the 96-well plates, as bacteria can settle at the bottom. Each 96-well plate was divided into four groups as follows: control group (bacterial suspension without 4I or light), 4I alone group (bacterial suspension with 4I but no light), light alone group (bacterial suspension with light but no 4I) and PACT group (bacterial suspension with 4I and light). The 4I alone group and the control group were covered with aluminium foil and incubated for the same illumination time as the PACT group and the light alone group. After illumination, each well was serially diluted 10-fold in PBS to 10^5^ times, and 100 μl aliquots were then taken from every dilution to streak horizontally on sterile LB agar plates, which were incubated in the dark for 18 h at 37°C. The photoinactivation abilities of 4 groups against the two types of *A*. *baumannii* strains were evaluated by minimum inhibitory concentration (MIC) and minimum bactericidal concentration (MBC) values. The MBC was defined as the concentration corresponding to less than 5 colonies on the agar plates, and the MIC was defined as the concentration corresponding to only slight growth of bacteria could be observed on the agar paltes.

### Dark toxicity assay *in vitro* with Balb/c 3T3 fibroblasts

Balb/c 3T3 fibroblasts were used in the dark toxicity and phototoxicity assay studies of 4I *in vitro*. Briefly, Balb/c 3T3 fibroblasts were cultured in 60 mm tissue culture dishes, grown in Dulbecco’s modified essential medium (DMEM; Gibco, USA) with 10% heat-inactivated fetal bovine serum (FBS; Gibco, USA), penicillin (1000 units/mL) and streptomycin (100 g/mL), and incubated at 37°C in a 5% CO_2_ atmosphere humidified incubator. When the cells reached 80% confluence, they were washed with PBS, trypsinized with 1 mL of 0.25% trypsin-EDTA solution, centrifuged and measured by haemocytometer for experimental use. Then, cells were seeded in a 96-well plate with a density of 10^5^ cells/well (100 μl total volume/well) and incubated for 24 h for cell adherence. The 96-well plates were divided into 3 parts with PACT, 4I alone and control groups. On the following day, double dilutions (0.975–125 μM) of the 4I solution were prepared in DMEM, and the cells were washed with PBS again. Then, 100 μl aliquots of gradient concentrations of 4I or DMEM were added to the corresponding samples for 30 min incubation in an incubator. The PACT sample was illuminated with the same light source for 30 min as described above. The 4I alone sample was covered with aluminium foil and incubated for 30 min as well. After illumination, the samples were stored in the incubator for 24 h. The next day, examination of cell viability was performed at 4-h using an absorption wavelength of 450 nm and the WST-8 assay with a colorimetric cell counting kit (Dojindo, Japan). Similarly, the half lethal concentration (IC_50_) and its 95% confidence interval (CI) was calculated by SPSS software.

### Full-thickness excisional wound model infected with *A*. *baumannii* and photodynamic treatment *in vivo*

The animal experiment protocol was approved by the Subcommittee on Research Animal Care of the Affiliated Hospital of Logistic University of Chinese People’s Armed Police Force (PAPF), and all animal procedures were carried out according to the National Institutes of Health guidelines on the use of experimental animals. Adult female BALB/c mice (purchased from the Experimental Animal Center at the Academy of Military Medical Sciences) that were 6–8 weeks old and weighed 18–20 g were used in this study. The animals were kept one per cage with access to food and water ad libitum at a temperature of 21–25°C and a relative humidity range of 50% to 60%. A 12-hour light-dark cycle was provided at the same time. The mice were acclimatized for one week prior to experimentation and were excluded if they lost weight during this period. The full-thickness excision wound model was performed referring to published papers [14–16]. Briefly, mice were randomly divided into five groups of six animals each, then these groups were designated as (A) non-infected group; (B) non-treated infected group; (C) 4I alone treated infected group; (D) light alone treated infected group; (E) PACT treated infected group. At days 1 and 4 before modelling, all mice were intraperitoneally injected with 150 mg/Kg and 100 mg/Kg of cyclophosphamide (C106991, Aladdin, USA) respectively, to increase mice susceptibility to infection. Mice were anesthetized by injecting with 0.5% (0.012 mL/g) pentobarbital sodium (Merck, Germany) and then shaved to make an approximate 1 cm × 1 cm wound on their dorsal surfaces. The wounds did not cut through the panniculus carnosus and had no visible bleeding. Mice in group A were put into cages as described previously. Five minutes after wounding, an aliquot of 50 μl of a suspension containing 10^8^/mL CFU multidrug-resistant *A*. *baumannii* in PBS was inoculated on the other 4 groups. 30 minutes interval was a prerequisite according to the outcome of our study group, during which the bacteria could attach to the tissue and the PS could be taken up by bacteria at maximum levels [9]. 50 μl of 4I solution (40 μM in PBS) was applied to C and E groups, and the placebo (PBS) was added to the B and D groups. At last, the D and E groups were illuminated with a 650 nm laser at a fluence of 50 J/cm^2^.

#### Survival, body weight and wound healing follow-up

Mice were humanely euthanized in moribund condition (poor food intake, inactivity, ambulation difficulty, flexuous posture, shivering, ruffled fur, 15% reduction in weight) or at the fixed time point of post-infection. The performance status of mice with full-thickness excisional wound infected with 10^8^ CFU multidrug-resistant A. baumannii were observed and recorded on a daily basis. Humane euthanasia was achieved by gradually replacing atmosphere air with CO_2_ in a closed container. Wound areas were measured every three days until the wounds had healed. Mice were held on a plat in the prone position with a ruler as a length reference standard. The photographs of wounds were taken by a digital camera, which was positioned just above the mice.

#### Cytokine analysis of inferior vena cava blood serum

Mouse were anesthetized at 1, 4, 7 and 10 d after infection by the same method as mentioned above and placed in the supine position. One millilitre syringes were used, and inferior vena cava blood was collected in anticoagulant tubes temporarily. Serum was obtained by centrifugation and preserved at -80°C. The concentrations of basic fibroblast growth factor (bFGF), tumour necrosis factor (TNF)-α and interleukin (IL)-6 in the serum were determined with a mouse bFGF ELISA kit (Abcam, UK), a mouse proinflammatory cytokine TNF-α ELISA kit (eBioscience, USA) and an IL-6 ELISA kit (eBioscience, USA) according to the manufacturers’ instructions.

#### Bacterial burden of wound tissues

Once inferior vena cava blood was collected, mice were sacrificed with CO_2_. Wounds were taken sterilely prior to removal, and each wound tissue was placed in a sterile tube containing a given amount of PBS (9 times the tissue weight). Then, the tissues were minced to release the bacteria. This suspension was serially diluted 10-fold in PBS to give dilutions of 10^−2^ to 10^−5^ times, in addition to the 100 μl aliquots of original concentration. Each of the dilutions were streaked horizontally on sterile LB agar plates and incubated in the dark for 18 h at 37°C. Surface bacterial load was counted manually.

#### Histological examination of wound tissue

To evaluate the effects of different protocols on the healing process of the infected wounds at histological levels, wounded and other visceral tissues including kidney, liver, lung and heart from mice of different groups were surgically excised at 1, 4, 7 and 10 days post wounding, and these specimens were preserved in 10% neutral buffered formalin for more than 24 h before being dehydrated in alcohol, cleared in xylene and embedded in paraffin. Serial 4-μm-thick tissue sections were obtained and stained with haematoxylin and eosin (H&E). A pathologist blinded to the study observed the morphological changes, such as inflammation, presence of capillaries, arrangement of collagen fibres and reepithelization.

### Statistical analysis

All statistical analyses were performed with SPSS software version 22.0. Data were expressed as the mean ± standard error. Wound area calculations were performed by Image J software. When the data conformed to homogeneity of variance, the LSD method was used. Otherwise, non-parametric Tamhane’s T2 was used. Significance was set at P<0.05.

## Results

### Photodynamic inactivation of bacteria *in vitro*

**Fig 2:**
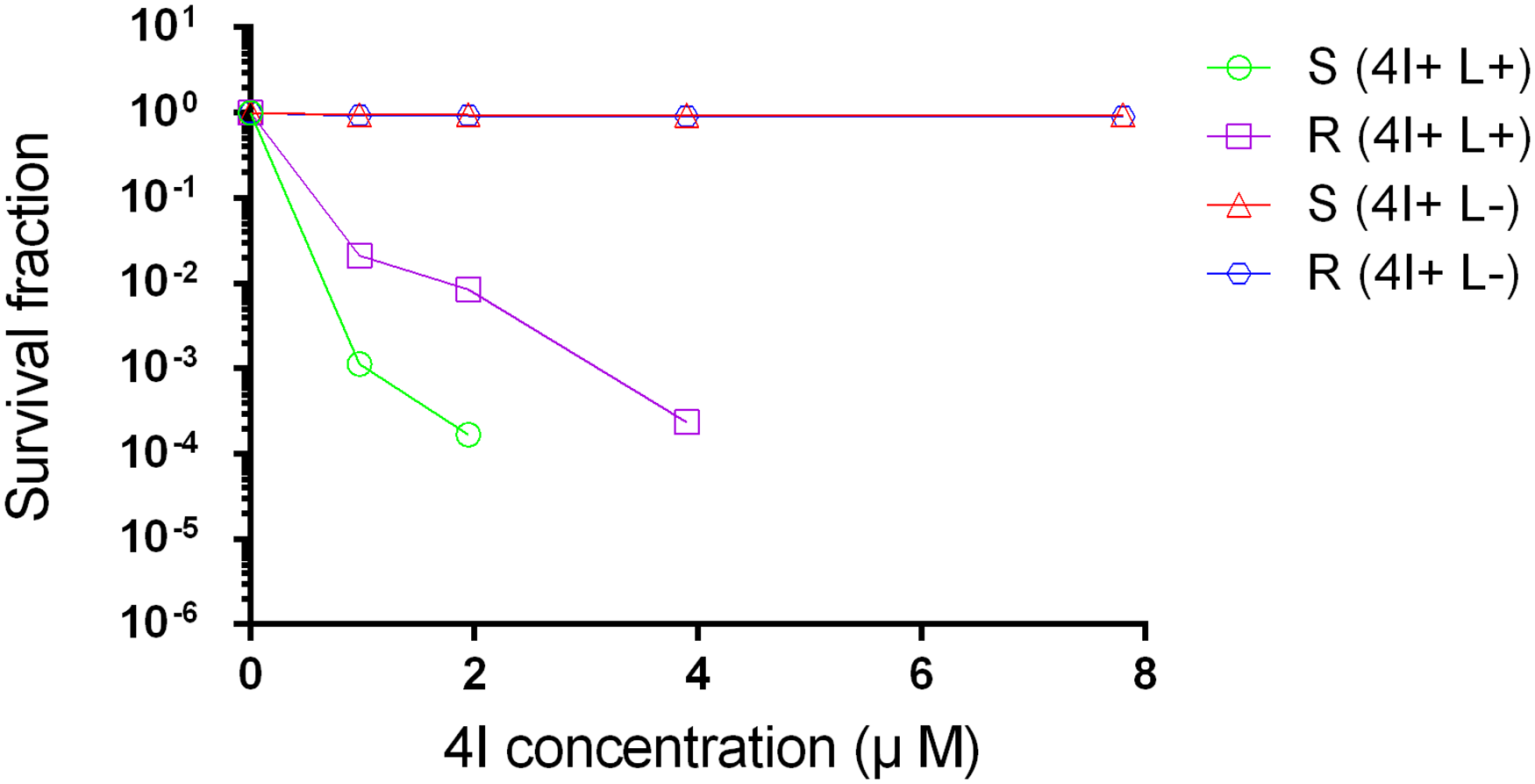
Survival fraction of two strains treated by different concentrations of 4I with or without illumination (6J/cm^2^ for 30 min). S, sensitive strain; R, multidrug strain; 4I+ L+, treated by 4I combined with light; 4I+ L-, treated by 4I not combined with light.

The photodynamic activity of compound 4I for eliminating both a clinically isolated multidrug-resistant and a relatively sensitive *A*. *baumannii* strain was tested under *in vitro* conditions by calculating the MBC and the MIC, and which was carried out in triplicate. The mean values of MBC and MIC of the multidrug-resistant and relatively sensitive strains in PACT and 4I alone groups are shown in Table 1. The multidrug-resistant and relatively sensitive strains had high susceptibilities to compound 4I under illumination with MBC values of 3.9 μM against the sensitive strain, where the sensitive strain was eradicated thoroughly with no visible colonies on LB plates, and 7.8 μM against the multidrug-resistant strain, where less than five colonies on a LB plate. The survival fraction curves indicating the correlation between concentration-dependent and photoinactivation effects for the two strains with or without light conditions are presented in Fig 2. A significant difference on decrease of bacterial load between the PACT group and the 4I alone group (P<0.01) and the correlation between increasing concentration and the declining bacterial survival fraction in the PACT group were observed. For the sensitive strain, compound 4I with illumination achieved approximately 2.89 log and 3.83 log unit reductions in the bacterial survival fraction at the concentrations of 0.975 μM and 1.95 μM, respectively. Other serial concentrations equalling or exceeding the MBC are not shown in this figure, as the descendent bacterial survival fractions were all 6 log_10_. The multidrug-resistant strain in the PACT group induced bacterial inactivation by 1.79 log, 2.15 log, and 3.77 log unit reductions with concentrations of 0.975 μM, 1.95 μM, and 3.9 μM, respectively. From the values of MIC, MBC and decreased bacterial load, we can conclude that PACT mediated by 4I showed higher bactericidal activity on the sensitive *A*. *baumannii* strain than on the multidrug-resistance strain (P<0.01). Nevertheless, in the no light group, 4I did not produce significant growth inhibition on either the sensitive or multidrug-resistant strains across all concentrations. In addition, light alone groups did not inhibit the growth of the two strains.

**Table 1:** Minimum bactericidal concentration (MBC, μ M) and minimum inhibitory concentration (MIC, μ M) of 4I against multidrug-resistant and sensitive strain under with or without light conditions.

*A*. *baumannii* strain	PACT groups		4I alone groups	
	MBC	MIC	MBC	MIC
multidrug-resistant (R)	7.8	3.9	>125	>125
relatively sensitive (S)	3.9	1.95	>125	>125

### Comparison of cell viability in Balb/c 3T3 between PACT and 4I alone groups

**Fig 3:**
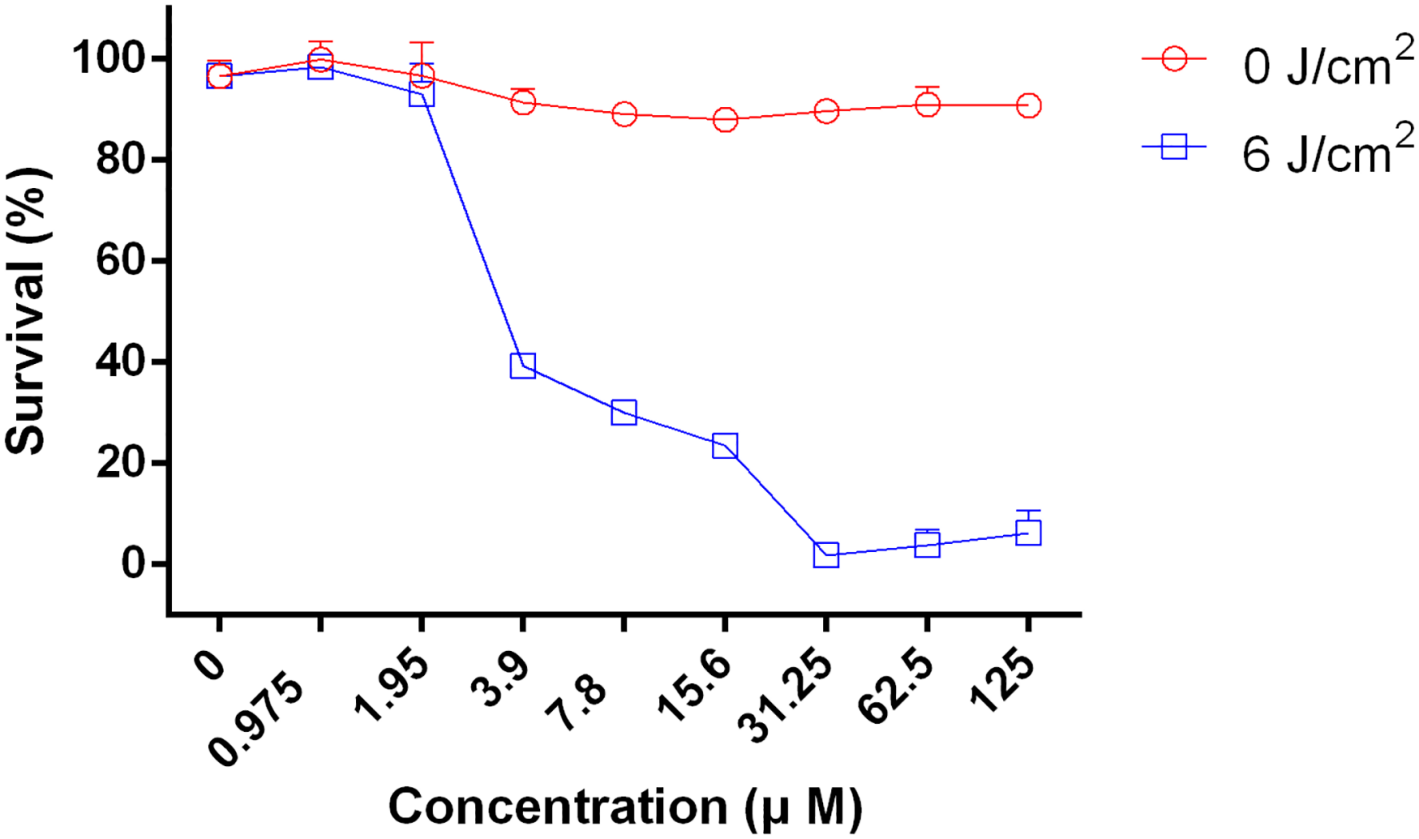
The cell viability of Balb/c 3T3 fibroblasts combined 4I of different concentrations with or without illumination determined by CCK-8 assay.

The dark toxicity and phototoxicity of 4I against Balb/c 3T3 fibroblasts were investigated by CCK-8 assay for different concentrations, and which were repeated three times. As shown in Fig 3, the cell viability reached approximately or above 90% in the range of 0 to 125 μM without illumination conditions, and significant differences were not obtained among different concentrations (P>0.05). The cell viability was also over 90% in the range of 0 to 1.95 μM after illumination with 6 J/cm^2^; nevertheless, the cell viability sharply dropped to lower than 40% at 3.9 μM. At 1.95 and 3.9 μM, the MICs of the sensitive and multidrug-resistance strain, less than 20% and 40% cells were dmaged, respectively. At 3.9 μM, the cell viability dropped sharply, nearly all of the sensitive strain and more than 93% of the multidrug resistance strain were killed. These results indicated that *A*. *baumannii* strain suffered more serious damage compared with cells under the same concentration conditions.

### Survival and body weight

**Fig 4:**
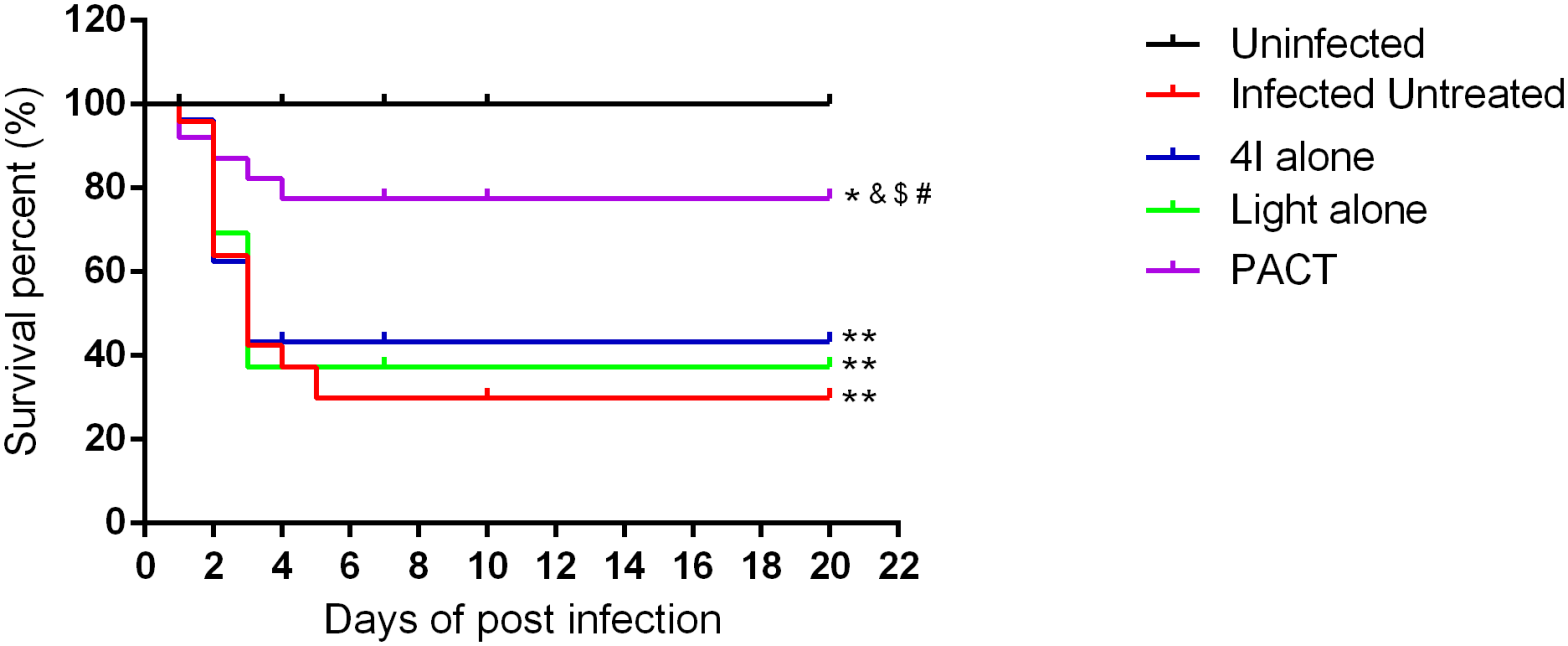
Survival curves for the uninfected and the infected *A*. *baumannii* mice treated by different treatments during the time courses (from day 0 to complete healing). * indicates significance vs. uninfected group (*P<0.05, **P<0.01), & indicates significance vs. no treatment group (^&^P<0.05), $ indicates significance vs. 4I alone Group (4I + light -, ^$^P<0.05), # indicates significance vs. light alone group (4I –light +, ^#^P<0.05).

The performance status of mice with full-thickness excisional wound infected with 10^8^ CFU multidrug-resistant *A*. *baumannii*, including the food intake, body weight, activity and survival, were observed and recorded on a daily basis. Each group of mice presented with inactivity, lost weight within 72 h after establishing the model; however, non-treated mice markedly presented with these problems, leading to a moribund condition, and death often occurred between 48 h and 72 h post-infection. Fig 4 shows the survival curves for the 5 different groups, and it demonstrates that the mice treated by PACT had a noticeable survival advantage over the non-treated, the 4I alone treated and the light alone treated mice. The survival rates of the A, B, C, D and E groups were 100.00%, 45.83%, 52.00%, 55.56% and 80.00%, respectively, and statistical significance was observed. Variations in mean body weight were obtained from the five different groups mice, and the results showed that the maximum weight loss occurred at day 1 post-model establishment among all groups mice; only weights from the non-infected and PACT groups returned to normal at day 3; the other three groups of mice (light alone group, 4I alone group, and non-treated group) needed more than 4, 5, and 8 days, respectively.

### Bacterial load in full-thickness excisional wound with *A*. *baumannii*

**Fig 5:**
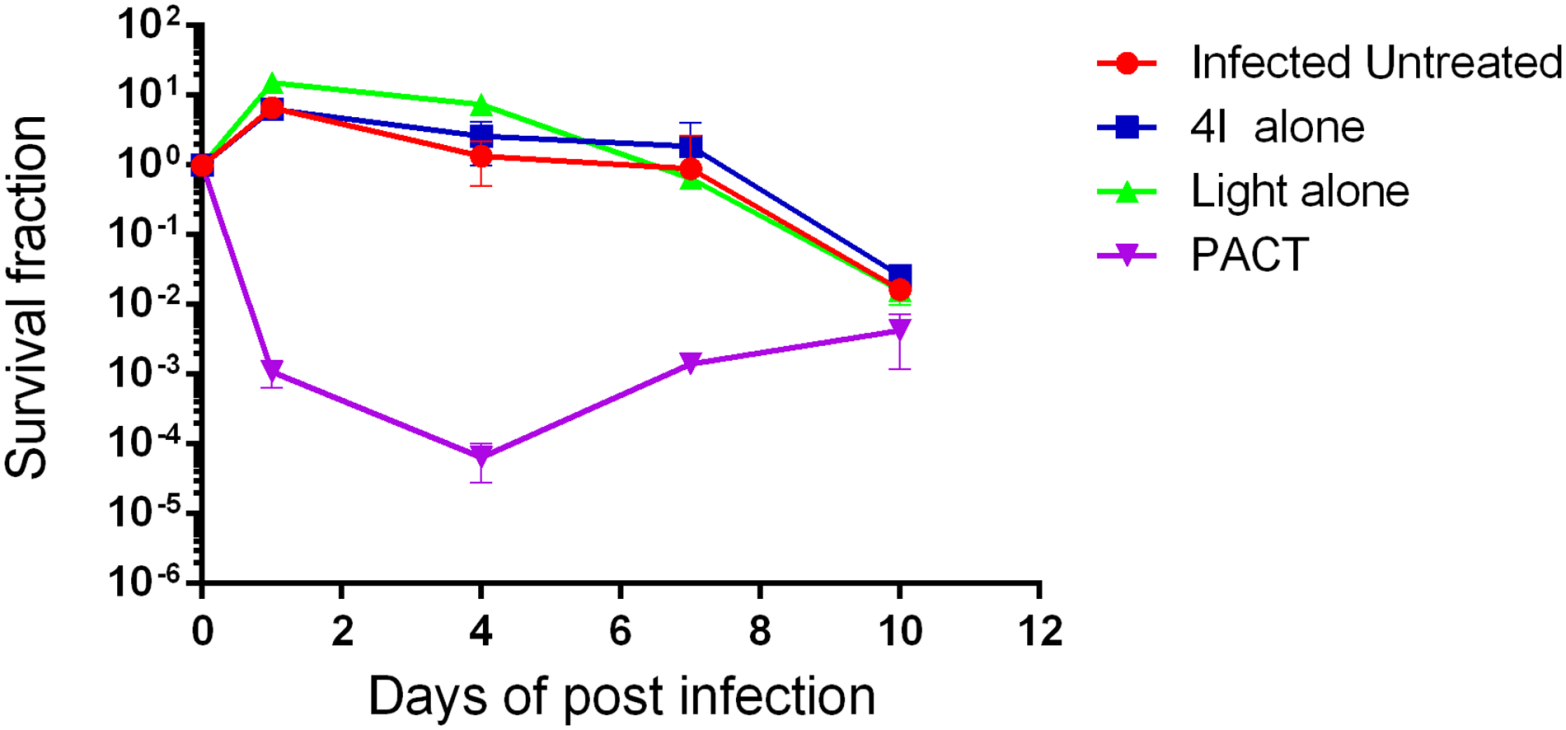
Survival fraction of *A*. *baumannii* isolated from excised tissues of mice in four different treatment groups at determined time points.

The promise in eliminating *A*. *baumannii*, a clinically isolated multidrug-resistant strain, mediated by 4I followed up irradiation (650 nm, 6 J/cm^2^) *in vitro*, promoted further investigations on wounds infected with *A*. *baumannii* in mice *in vivo*. The antimicrobial photoactivity was evaluated by quantitative bacterial count on agar plates, where homogenates from the excised tissues at different time points post treatment were streaked. As shown in Fig 5, the survival fraction of *A*. *baumannii* isolated from the skin excision wounds treated with 40 μM 4I and a fluence of 50 J/cm^2^ was significantly reduced compared to the other three groups in the following days after different treatments. The PACT group induced a 2.89 log decrease in bacterial counts, while the infected non-treated and 4I alone treated groups caused an increase of approximately 7-fold, and the light alone treated group had a 16-fold increase at day 1 post treatment. The bacterial count in the non-treated, 4I alone and light alone groups differed significantly from the PACT group (P = 0.011, 0.034 and 0.021); furthermore, the non-treated and 4I alone groups differed significantly from the light alone group (P = 0.011 and 0.034). At day 4, the PACT group presented a 3.94 log reduction, and the bacterial burden in the other three groups had negligible reductions but were still higher than the initial incubation number during the same time. Significant differences (P<0.05) were observed between the other three groups and the PACT group. Bacterial re-growth was observed in the PACT, non-treated and 4I alone groups at day 7 after different treatments. Although the reduction of the bacterial level was 2.86 log in the PACT group, it was kept almost 3 log lower than the other three groups, and all the differences were significant (P<0.05). In terms of results at day 10 post-infection, the bacterial burden of the PACT group was still visually lower than the other three groups; nonetheless, the advantage in eradicating *A*. *baumannii* conducted by 4I and irradiation disappeared.

### Wound healing

**Fig 6:**
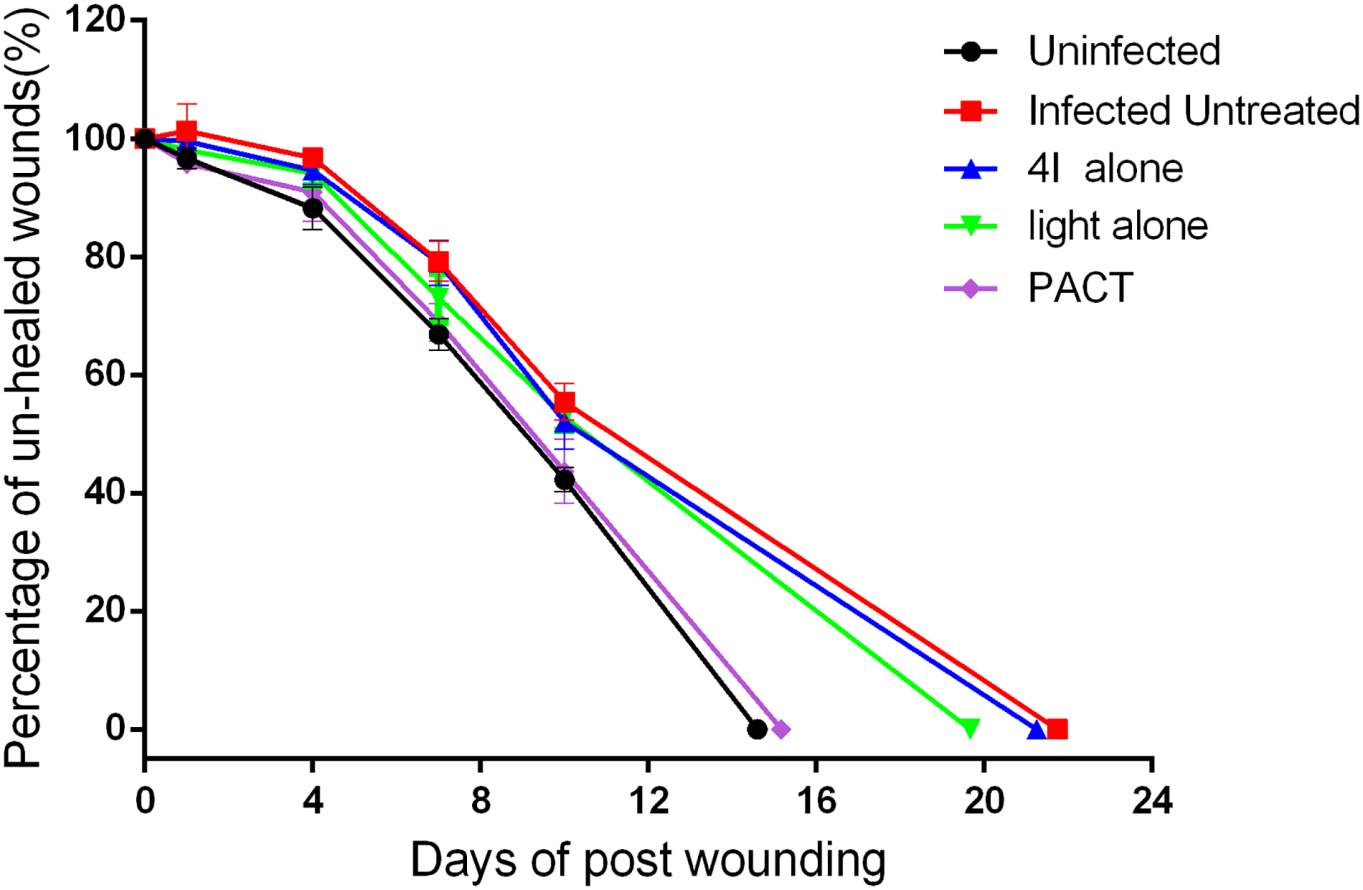
The percentage of un-healed wound areas for five different groups.

The wound healing rate and the time of the wound to be completely healed are significant indicators to evaluate the therapeutic effect of PACT compared with the other three treatments. Fig 6 shows the un-healed rates of the full-thickness excisional wounds of all the groups studied. Compared with the other four groups, a small increase in wound size within 2 days post-infection was found in the non-treated group. At day 1, the healing rates of the non-infected group and the PACT group were 3.33% and 4.24%, respectively. At day 1 after the experiment, the healing rate of the non-infected group differed significantly from the non-treated and the 4I alone groups (P = 0.028 and 0.018, respectively); the PACT group differed significantly from the non-treated, the 4I alone and the light alone groups (P = 0.025, 0.010 and 0.018, respectively). At day 4, the healing rate of the non-infected group differed significantly from the non-treated, the 4I alone and the light alone groups (P = 0.006, 0.016 and 0.028, respectively); the PACT group differed significantly from the non-treated group (P = 0.01). On day 7, the non-infected group differed significantly from the non-treated, the 4I alone and the light alone groups (P = 0.001, 0.002 and 0.048, respectively); the PACT group differed significantly from the non-treated and 4I alone groups (P = 0.004 and 0.007, respectively). Similarly, some significant differences were observed at day 10. The non-infected group differed significantly from the non-treated, the 4I alone and the light alone groups (P = 0.004, 0.034 and 0.004, respectively); the PACT group differed significantly from: the non-treated, 4I alone and light alone groups (P = 0.031, 0.034 and 0.05, respectively). As the above statistical analysis shows, the PACT group accelerated healing of excisional wounds infected with *A*. *baumannii* by destroying bacteria, and the advantage was marked from the seventh days. In addition, the average wound healing times of the non-infected, non-treated, 4I alone, light alone and PACT groups were 14.6±1.14, 21.75±1.50, 21.25±1.71, 19.67±2.08 and 15.17±0.98 days, respectively. Significant differences between the groups of non-infected and non-treated, 4I alone, light alone (P = 0.014, 0.014 and 0.024, respectively) were observed; moreover, the PACT group differed significantly from the non-treated, 4I alone and light alone groups (P = 0.009, 0.009 and 0.018, respectively).

### Cytokine expression levels analysis

**Fig 7:**
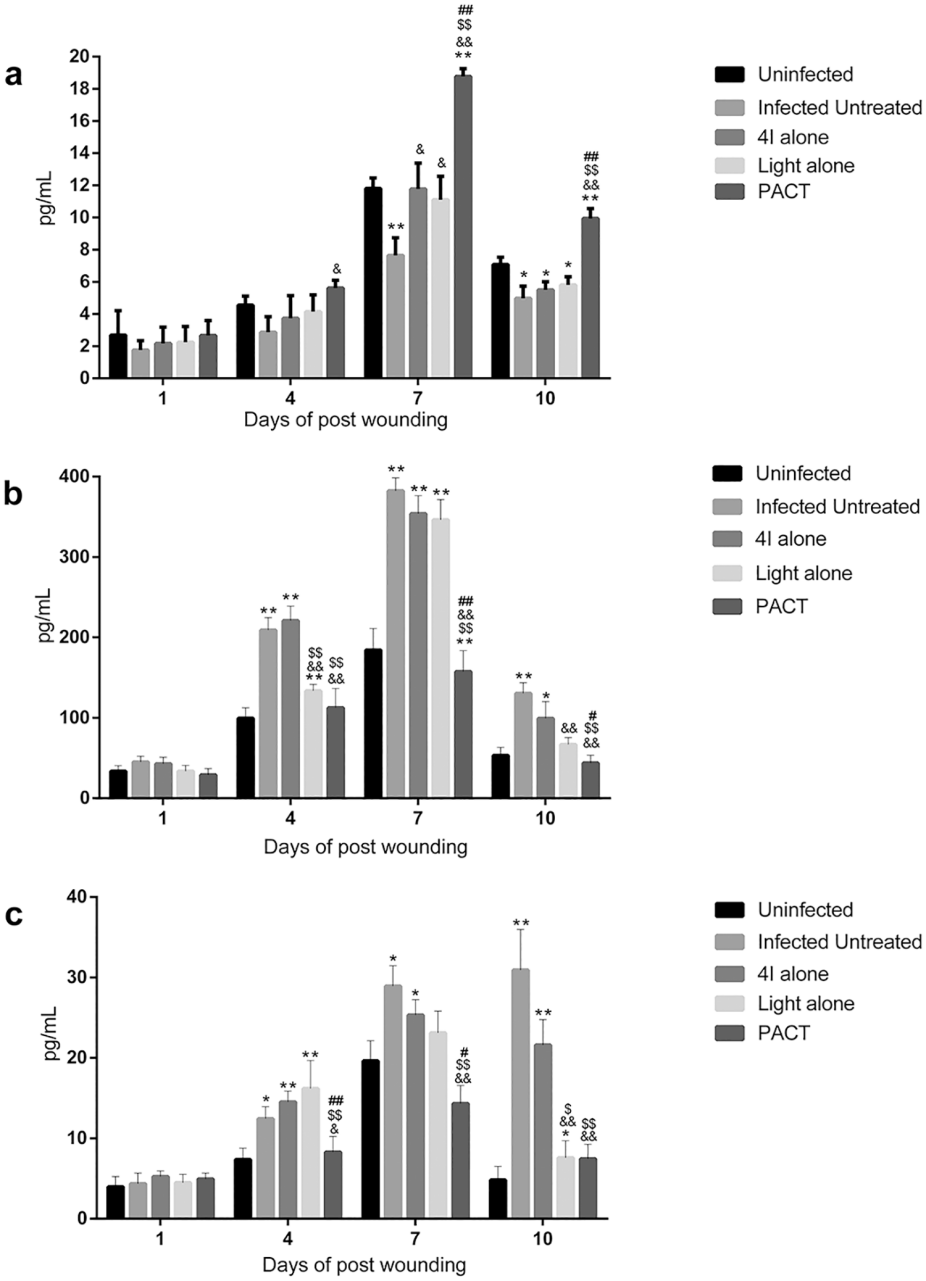
The serum expression levels of growth factor and pro-inflammatory cytokines for different treatment mice at multiple time points. (a) basic fibroblast growth factor, bFGF; (b) interleukin (IL)-6, IL-6; (c) tumor necrosis factor (TNF)-α, TNF-α. The results are presented as mean±SE. * indicates significance vs. uninfected group (*P<0.05, **P<0.01), & indicates significance vs. infected untreated group (^&^P<0.05, ^&&^P<0.01), $ indicates significance vs. 4I alone group (4I + light -, ^$^P<0.05, ^$ $^P<0.01), # indicates significance vs.light alone group (4I –light +, ^#^P<0.05,^##^P<0.01).

To gain further insight into the immunomodulatory and healing effects of various treatments on the experimental full-thickness excisional wound with or without infection, serum samples were collected and subjected to three ELISA analyses as described above. Fig 7 shows the bFGF, IL-6 and TNF-α expression levels in serum at different time points post-wounding. The bFGF, IL-6 and TNF-α serum levels were not statistically significantly different among the different groups at day 1 post-wounding because of the short interval and immunosuppression. The secretion levels of the three cytokines peaked within 7 days of wounding, and statistical significance was noted among the different protocols. In general, the pro-inflammatory cytokine (IL-6 and TNF-α) levels in infected mice treated with 4I combined with a light dose of 50 J/cm^2^ decreased, and the growth factor (b-FGF) increased significantly.

### Histological analysis

**Fig 8:**
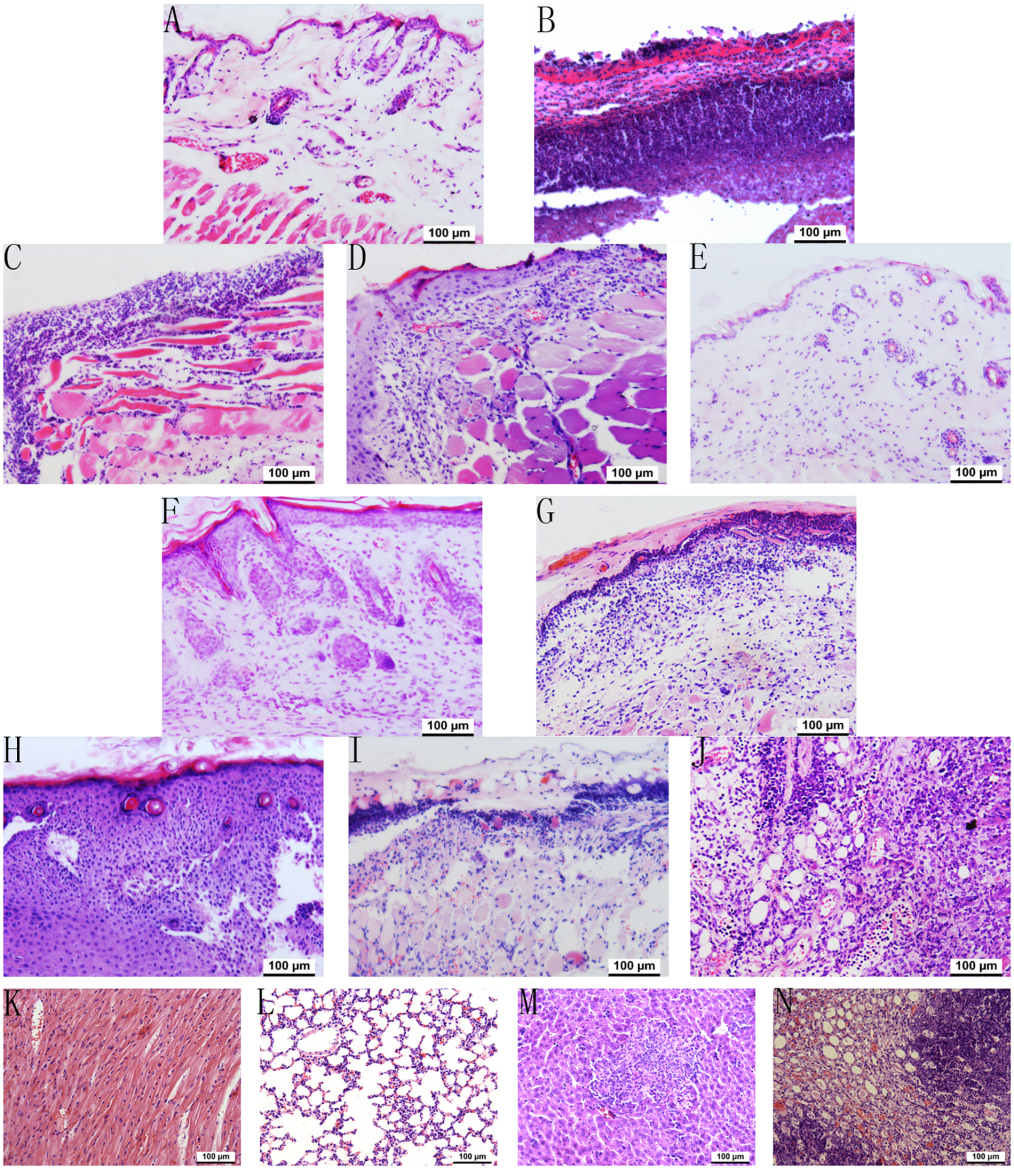
Haematoxylin & eosin stained sections of the wound skin and other organs. (A-E) the wound skin at day 4: (A) uninfected group; (B) infected untreated group; (C) infected 4I alone treated group; (D) infected light alone treated group; (E) infected PACT treated group (magnification 200×). (F-J) the wound skin at day 7: (F) uninfected group; (G) infected untreated group; (H) infected 4I alone treated group; (I) infected light alone treated group; (J) infected PACT treated group (magnification 200×). (K-N) the other organs at day 4 in infected untreated group: (K) heart; (L) lung; (M) liver; (N) kidney (magnification 200x).

The therapeutic effects of the five different modalities were assessed and obtained through observing the morphological features during the healing progress under a light microscope. At day 1 after wounding, biopsies from the groups of non-infected, non-treated, 4I alone, light alone and PACT did not exhibit a clear demarcation in terms of the pathological representation. At day 4, the histological appearance of the wound repair process showed a degree of demarcation among the five different treatment regimens (Fig 8A–8E). Infiltration with a marked number of inflammatory cells, oedema and a defect in the epithelium appeared more commonly in the non-treated, 4I alone and light alone groups than in the non-infected and PACT groups. Bacterial colonies in the tissue were observed, particularly in these three groups. An initially thin granulation tissue poor in fibroblasts and microvessels and a continuous epithelial lining were shown in the uninfected group, which exhibited a degree of wound remodelling comparable to that of the other four different regimens administered to the mice. Otherwise, the PACT group showed a marked reduction in inflammatory cells. The histological appearance of the wound repair process showed that the wound remodelling was more advanced a week later, but the non-infected and PACT-treated mice performed better in the wound closure period, shown as a wide zone of granulation tissue with numerous vessels, abundant inflammatory cells and fibroblasts, and moderate deposition of collagen fibres in the wound’s edge and bed area (Fig 8F–8J). Conversely, infected mice not receiving treatment or receiving 4I alone and light alone treatments exhibited a quite different and delayed wound-healing process: thinner granulation tissue presented in most of the cases, with scarce vessels and fibroblasts, robust inflammatory response, and reduced collagen deposition. Abscesses were occasionally observed in the non-treated infected mice through all experimental periods. Similarly, the degree of damage to visceral tissues such as kidney, liver, lung and heart seemed to be related to the type of treatment. That is, the damage was the most serious in the non-treated infected mice compared with the other groups. The histological results revealed that kidney and liver were the major affected organs, in which infiltration with massive inflammatory cells, abscesses and necrosis occurred more commonly (Fig 8K–8N).

## Discussion

*Acinetobacter baumannii* has caused a continuously rise in nosocomial infections, and has been considered as a “serious” threat by the Centers for Disease Control and Prevention [17, 18]. The high degree of adaptation to the environment, inappropriate empiric therapy and own genetic characterization mutations have made the *A*. *baumannii* resistant to antibacterial agents, particularly to carbapenem. In addition to the paucity in development of novel antibiotics, antimicrobial therapy has been a difficult clinical conundrum [2, 19, 20]. Whereas photodynamic antimicrobial chemotherapy has high efficiency against microorganisms, non-selection and difficulty in producing drug resistance has been proposed as a potential alternative treatment for localized infection induced by drug-resistant pathogens [21]. In this study, the lysine-porphyrin conjugate 4I, a new class of PSs, was employed, and the efficacy of PACT *in vitro* for photo-inactivating *A*. *baumannii* isolated from clinical patients and *in vivo* for wound healing in full-thickness excisional wound model, with the combination of red light, was investigated. Our team has performed much work on the synthesis, characterization, and mechanism study of conjugate 4I [9, 22]. It is gratifying that 4I displays some advantages including high quantum yields of ^1^O_2_ production (Φ_Δ_ = 0.95), good biocompatibility and water-solubility compared to other porphyrins bearing unnatural or natural cationic groups [9, 13, 23–25].

In general, 4I-mediated PACT was highly effective in photoinactiving bacteria, but the presence of light and a photosensitizer were indispensable, which also conformed to our knowledge. Furthermore, there was a difference in sensitivity to two different *A*. *baumannii* strains, one multidrug-resistant and one antimicrobial-sensitive. This results are similar to the published paper, which shows MRSA are significantly more resistant to photoinactivation than MSSA strains [26]. The sensitivity difference could be attributed to the drug-resistance mechanism of *A*. *baumannii* strain. The formation of drug-resistance mechanisms are related to drug inactivation or alteration, modification of drug binding sites, porin loss, efflux pumps and biofilm formation. However, experts favour the efflux pump mechanism [27]. Efflux systems are classified into six super-families based on their sequence similarity, and the type of efflux pump of most Gram-negative bacteria belongs to the RND super-family, which can evade attacks of a variety of antibiotics and structurally unrelated molecules. Yet, a previous paper reported that the efflux pump mechanism does not itself confer high-level resistance but weakly increases the MICs, allowing bacteria to reach high-level resistance when associated with other mechanisms [28]. Previous study also showed the efflux pump mechanism of *S*. *aureus* could protect against photoinactivation mediated by methylene blue [29]. On the other hand, biofilm formation could lead to reduce susceptibility to photoinactivation [26]. ROS are formed after non-toxic photosensitizers are actived by irradiation with laser through either electron transfer (type I) or energy transfer (type II) mechanisms, which can react with many cellular components (cytoplasmic membrane, DNA/RNA, amino acids, unsaturated lipids and so on) and then cause leakage of cellular contents or inactivation of membrane transport systems and enzymes and last induce lethal damage to bacteria [30, 31]. Moreover, our colleague verified and validated the photoinactivation mechanism of 4I mediated PACT against MRSA, *P*. *aeruginosa*, *E*. *coli* and found it broke the bacterial membrane and wall, and then caused the leakage of genomic DNA [22]. These mean the photoinactivation mechanism of 4I-mediated PACT is similar to other photosensitizers and is the same between drug-resistant and drug-sensitive strains. The uptake levels between drug-resistant and drug-sensitive strains of the same bacteria should be analyzed further for our study team. Anyhow, the photoinactivation effects on two different sensitivity strains of the *A*. *baumannii* species conducted by 4I and laser irradiation was satisfying.

To advance the 4I-mediated PACT to the clinical study level, it is necessary to prove that PACT can eradicate bacteria selectively and does not cause severe damage to host tissues and cells. The results demonstrating that the dark-toxicity and the photo-toxicity of 4I against Balb/c 3T3 fibroblasts *in vitro* showed the 4I-mediated PACT treatment did not produce obvious dark-toxicity in all concentration grades and concentration-dependent differences upon illumination, which were similar to other reports [32, 33]. The cell viability decreased as 4I concentrations increased to or over 3.9 μM, which could be ascribed to the production of ROS. It have been proved that low concentration of ROS is essential for normal cellular processes, while high levels can induce deleterious effects on cells [30, 34]. Once increasing the PS concentration, the amount of PS bound to each cell increases; otherwise, photodynamic effects have a strong connection with photosensitizer binding due to the generation of ROS *in situ* [35]. Although the cell viability reduced dramatically when the concentrations of 4I were higher than 3.9 μM following a light dose of 6 J/cm^2^, we still believe that the deleterious effects on cells conducted by 4I-mediated PACT are weak, and the reasons are as follows: firstly, the 4I conjugate is a cationic porphyrin conjugate, which should preferably bind to Gram-negative bacteria because of the ionic interaction between the cationic porphyrin and the negatively charged lipopolysaccharides or peptidoglycans on bacterial cell walls [13]. On the other hand, the study demonstrated that PS uptake by mammalian cells was much more difficult than in bacteria because of the difference in the membrane structures between both types of cells [36, 37]. Above studies all suggested that cationic porphyrin photosensitizers binded to bacteria more easily than mammalian cells. secondly, the bacteria were photoinactivated mainly through oxidative damage caused by ^1^O_2_ in the cell outer membrane, cytoplasmic component, genomic DNA, etc., while its lifespan was of short duration (< 0.3 ~1.5 μs), which results in a very short diffusion distance (< 30–100 nm) when it reacted with biomolecules in the physical environment [38]. Otherwise, the decay of ^1^O_2_ is 5–10 time faster than formation [39]. These data suggested that ^1^O_2_ produced in the microbial cells was difficult to diffuse into the host cells. In other words, PS selectively bound to bacteria and did not have an effect on normal host cells. Moreover, the analysis of cell viability was performed under such situation, where only cells was presented. We speculate the cell viability will improve greatly if bacteria and cells incubate together in PACT group, and such study and its mechanism will also be our future works. The antibacterial efficacy of 4I-mediated PACT was tested on three common clinical pathogenic bacterial, MRSA, *Escherichia coli*, and *Pseudomonas aeruginosa*, *in vitro* respectively and on an excisional wound model infected with mixed bacteria *in vivo* [9, 22]. To develop a more serious infection animal model, temporary immunosuppression to reduce circulating neutrophils and the use of a highly virulent strain was necessary. In this study, we presented an immunosuppressive mouse model of a full-thickness skin excisional wound with multidrug-resistant *A*. *baumannii* infection to mimic the pathogenesis and process of wound healing in patients. Referring to a large amount of studies, 30 minutes infection protocol was performed in our study [14, 16, 32, 40–42]. Furtherore, bioluminescent strains were uesd in many papers, and bioluminescence images were taken immediately after bacteria were inoculated over each mice [14, 40–42]. Bioluminescence images showed that the bacterial inoculum applied to each wound consistently permeated into host tissue. Otherwise, the performance status of mice, survival rates of mice and levels of inflammatory cytokine in serum all showed the 30 minutes infection protocol could induce a real life infection.

Much higher light exposure or concentrations of photosensitizers were required for the *in vivo* than for the *in vitro* treatments because of competition with the exogenous photosensitizer between host cells and microorganisms [40, 43]. The working concentration *in vivo* was usually 5 or 10 times higher than that *in vitro*. Ten times of the MIC was chosen as the working concentration in this study [2, 44]. In this study, 20% mortality in the PACT treated group was observed, which was much less in the non-treated infected group, and the death in mice mainly occurred between 48 h and 72 h after infection. Moreover, the increase of bacterial burden that was also observed in the other three groups excludes the PACT treated group within 4 days post-infection.

The high mortality at days 2 and 3 and the bacterial increase within 96 h post-infection can be attributed to the bacterial growth phase, which is from day 1 to day 3, and the immunosuppression status of the mice. Bacterial rebound was also be reported in other papers and the trends of photoinactivation were similar between PACT groups and other groups although bacterial regrowth was present [16, 41, 45, 46]. The effect of photoinactivation on *A*. *baumannii* conducted by 4I-mediated PACT treatment could counteract the effect of bacterial regrowth. The results of *in vivo* antimicrobial experiments showed that the bacterial bioburden decreased most dramatically in the first phase (0–1 d) and moderately in the second phase (2–4 d), while bacterial numbers started to rebound in the third phase. Two factors were responsible for this regrowth. First, within 1 day after infection, the *A*. *baumannii* cells had not deeply penetrated into the tissues and remained superficial, which resulted in the easy inactivating of A. baumannii by ROS. However, at day 2 or day 3, survivals penetrated deep into tissue and multipled gradually; second, after 1 or 2 days, a biofilm would have formed due to the high biofilm-forming capacity of the *A*. *baumannii* strain [41]. Bacterial regrowth suggested that bacteria weren’t be eradicated entirely by PACT performed only once, and repeated applications of PACT were necessary to better counteract bacterial regrowth. Such study was rare in the current study with mouse models, and it will be our future work. Based on the gradual decline in bacterial number in the other three groups after the first phase, we presumed the temporary immunosuppression was slowly degenerating. Meanwhile, the innate immunity started to recover. Therefore, in the end, bacterial bioburden in all surviving mice from all groups nearly reached comparable levels.

Among the cytokines, basic fibroblast growth factor (bFGF), a multipotent growth factor, and a potent stimulant in mitoses and chemotaxis of mesodermal cells, such as fibroblasts and vascular endothelial cells, plays a pivotal role in the wound healing process [47]. Skin fibroblasts are the main repair cells and the major resource of extracellular matrix in the process of wound recovery. Ample evidences have demonstrated that interleukin-6 and tumour necrosis factor (TNF)-α are the pro-inflammatory mediators triggered by macrophages at the initial stage of inflammation and can suppress an excessive inflammatory response and apoptosis induced by trauma [48]. Therefore, the expression level of these mediators can act as markers to evaluate the healing rate and effect. In the ELISA analyses, the results broadly presented a trend that the 4I-mediated PACT group led to an increase in the expression of bFGF and a decrease in IL-6 with TNF-α compared to the non-treated infected group. The maintenance of low IL-6 and TNF-α levels in serum can contribute to the attenuation of excessive inflammatory responses regulated by the PACT regimen [49]. Studies have shown that exaggerated IL-6 levels can lead to an acute severe systemic inflammatory responses and subsequent sepsis because of the activation of the coagulation pathway and vascular endothelial cells along with the inhibition of myocardial function [50]. Similarly, the excessive TNF-α release is reported to induce apoptosis, inhibit angiogenesis and promote tissue degradation [16, 48]. That is, non-treated infected group mice subjected to higher inflammation, more severe degradation in tissues and microvessels and higher risk in dysfunction.

Indeed, the experimental results of the ELISA assays are strongly correlated and consistent with the wound healing rate, the healing time and the presentation of histological characteristics. The amount of bacterial bioburden in tissues, intensity of inflammatory responses, and level of wound recovery were all observed in the sections stained with H&E.

The data from wound healing rate and time analyses suggested that favourable wound repair was related to the reduction in bacterial load, shortened inflammatory phase and enhanced repair.

## Conclusion

In conclusion, the study not only demonstrated the efficacy of the PACT modality with the novel cationic lysine-porphyrin conjugate 4I and red laser in photoinactivating pathogenic bacteria, *Acinetobacter baumannii*, and the safety for host tissues and cells *in vitro* but also demonstrated the therapeutic effect for an excisional wound infected by a multidrug resistant strain *in vivo*. The encouraging results suggest that in addition to killing bacteria, 4I-mediated PACT will promote wound healing. Although synthesis, characterization of the conjugate 4I and evaluation of the mechanism of action of 4I-mediated PACT *in vivo* have been studied, more efforts and future clinical studies are warranted to deepen our understanding of PACT.

## Supporting information

S1 Fig Incubating multi-drug resistant strain in 4I alone group for 24h. It only has 4I photosensitizer.
(TIF)

S2 Fig Incubating multi-drug resistant strain in control group for 24h. It didn't has 4I photosensitizer nor light.
(TIF)

S3 Fig Incubating multi-drug resistant strain in PACT group for 24h. It has 4I photosensitizerand light.
(TIF)

S4 Fig Incubating sensitive strain in PACT group for 24h. It has 4I photosensitizerand light.
(TIF)

S5 Fig Incubating sensitive strain in 4I alone group for 24h. It only has 4I photosensitizer.
(TIF)

S6 Fig Incubating sensitive strain in control group for 24h. It didn't has 4I photosensitizer nor light.
(TIF)

S1 File The summary sheet of cell viability. (PDF)
