# Supplementary figures and images for: Photodynamic antimicrobial chemotherapy with the novel amino acid-porphyrin conjugate 4I: In vitro and in vivo studies
Source: PLoS One. 2017 May 11;12(5):e0176529. doi: 10.1371/journal.pone.0176529 (PMC5426629; doi:10.1371/journal.pone.0176529)

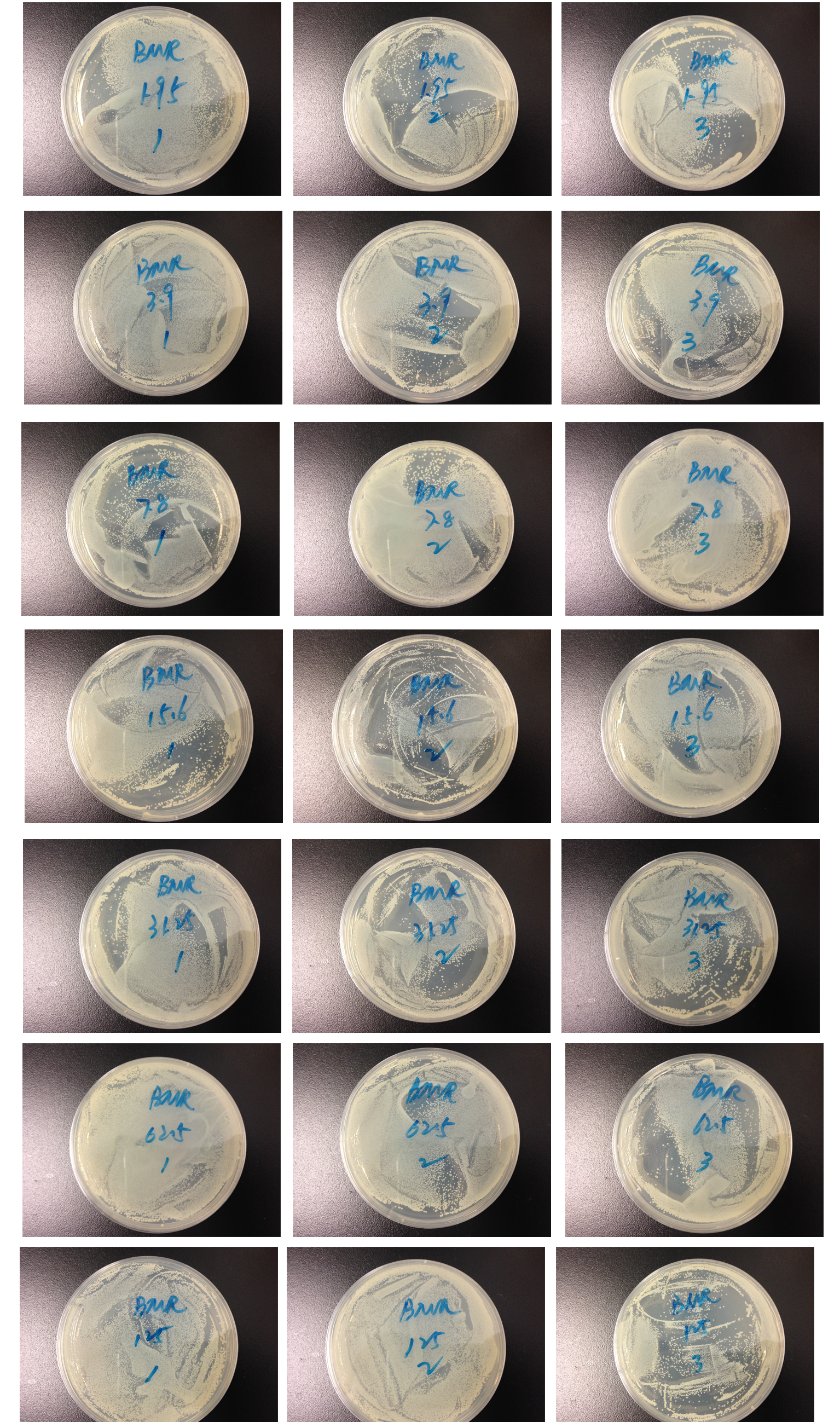

Supplement: S1 Fig — It only has 4I photosensitizer. (TIF) [file pone.0176529.s001.tif]

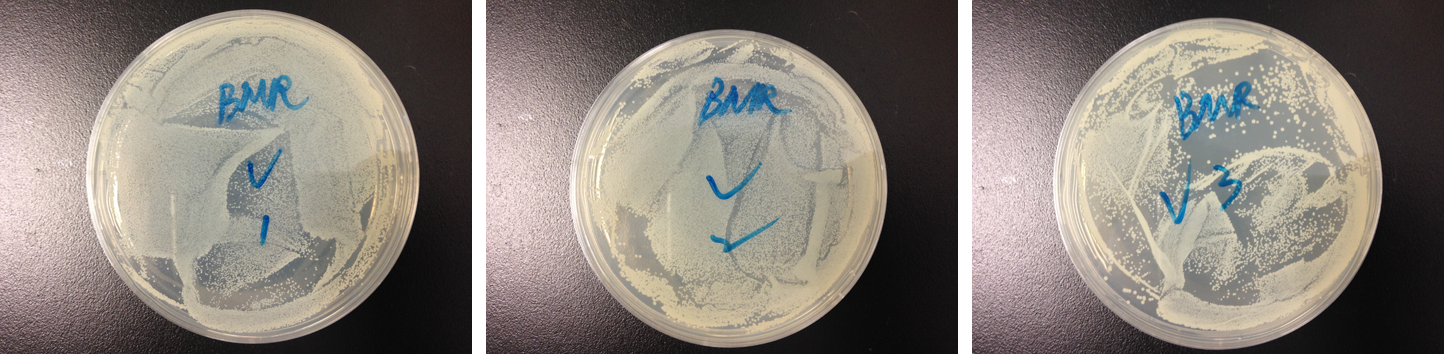

Supplement: S2 Fig — It didn't has 4I photosensitizer nor light. (TIF) [file pone.0176529.s002.tif]

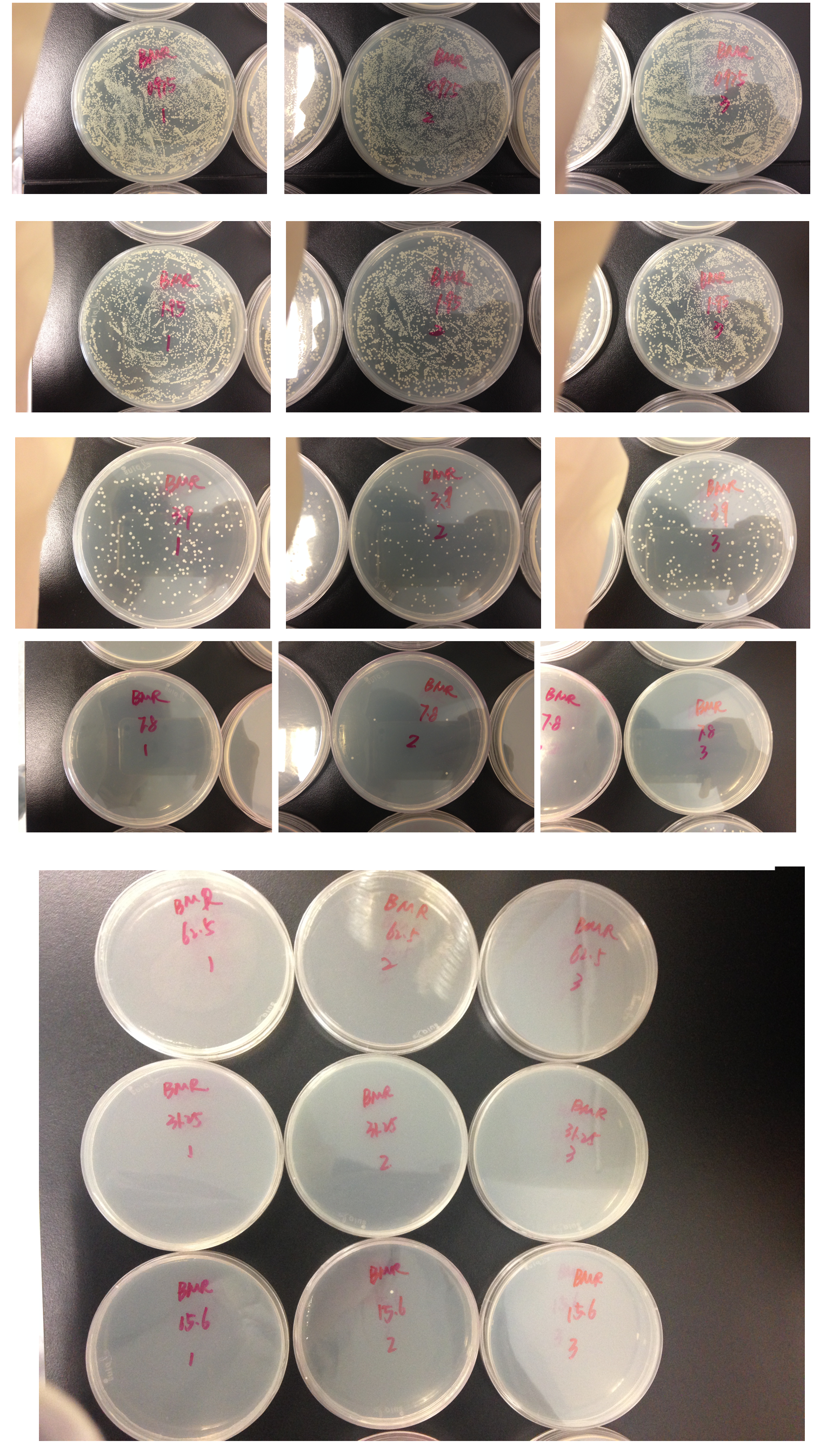

Supplement: S3 Fig — It has 4I photosensitizerand light. (TIF) [file pone.0176529.s003.tif]

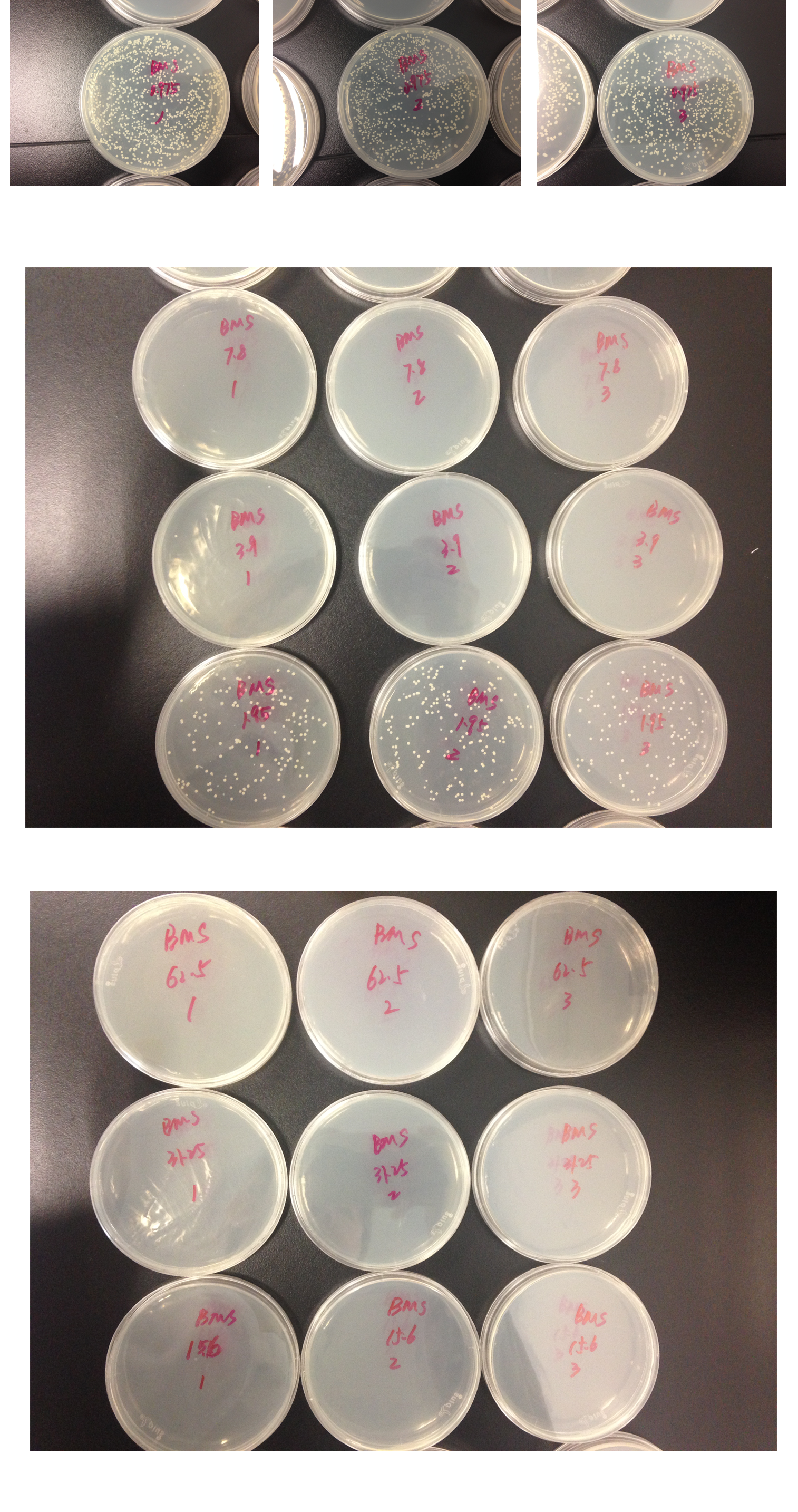

Supplement: S4 Fig — It has 4I photosensitizerand light. (TIF) [file pone.0176529.s004.tif]

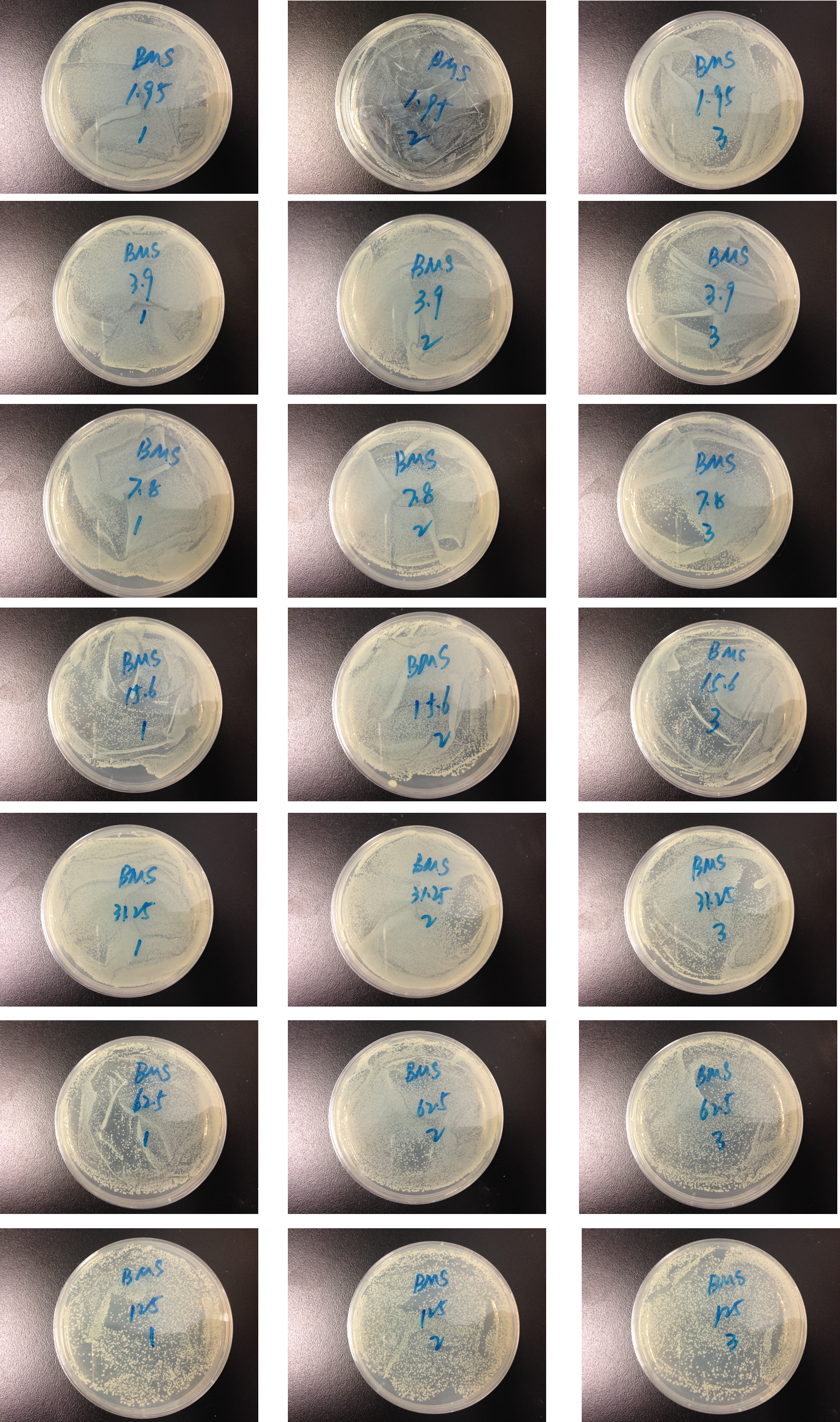

Supplement: S5 Fig — It only has 4I photosensitizer. (TIF) [file pone.0176529.s005.tif]

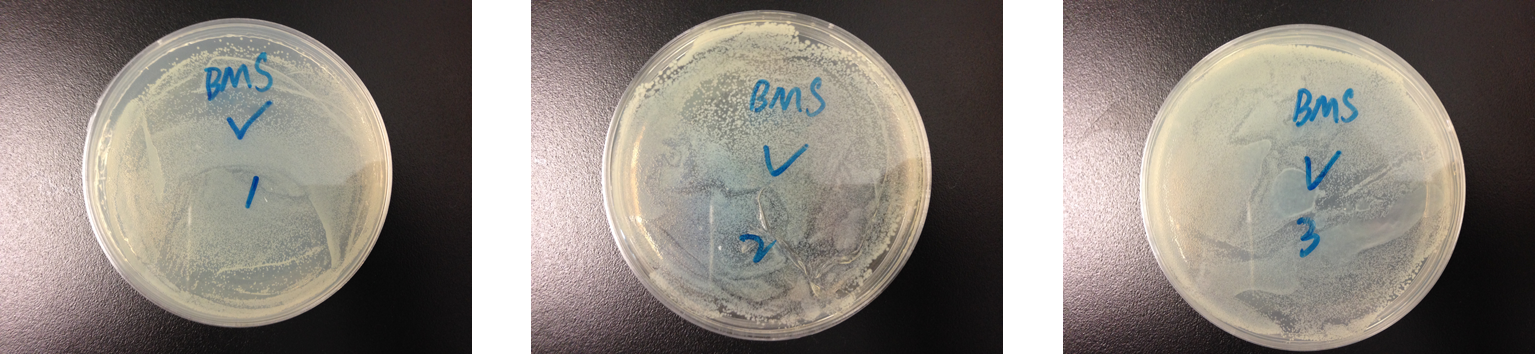

Supplement: S6 Fig — It didn't has 4I photosensitizer nor light. (TIF) [file pone.0176529.s006.tif]
