# Supplementary material for: Photodynamic antimicrobial chemotherapy with the novel amino acid-porphyrin conjugate 4I: In vitro and in vivo studies
Source: PLoS One. 2017 May 11;12(5):e0176529. doi: 10.1371/journal.pone.0176529 (PMC5426629; doi:10.1371/journal.pone.0176529)
Supplement: S1 File — (PDF) [file pone.0176529.s007.pdf]

PACT group

	0.975	1.95	3.9	7.8	15.625	31.25
	98.84%	97.40%	39.70%	30.06%	23.40%	1.55%
	97.43%	86.27%	38.19%	29.71%	23.00%	2.07%
	99.14%	95.53%	40.12%	30.28%	24.39%	1.81%
Mean	98.47%	93.07%	39.33%	30.02%	23.60%	1.81%
standard deviation	0.009098	0.059586	0.010153	0.002881	0.00717	0.002582

4I alone group	0.975	1.95	3.9	7.8	15.625	31.25
	97.35%	100.83%	93.36%	91.19%	87.41%	92.27%
	98.32%	89.23%	88.41%	86.94%	87.39%	87.47%
	104.13%	100.11%	92.58%	89.23%	89.27%	89.45%
Mean	99.93%	96.72%	91.45%	89.12%	88.02%	89.73%
standard deviation	0.03665	0.06501	0.026628	0.021294	0.01081	0.024131

Light alone group

	93.70%
	99.73%
	96.56%
Mean	96.66%
standard deviation	0.030149

Control grouop

	96.93%
	96.10%
	98.28%
Mean	97.10%
standard deviation	0.011003

62.5	125
2.64%	10.71%
1.53%	1.62%
7.24%	6.37%
3.80%	6.23%
0.030264	0.045457
62.5	125
91.02%	91.90%
87.60%	89.22%
94.63%	91.33%
91.08%	90.82%
0.035186	0.014108
